# Infertility improved by etanercept in ankylosing spondylitis

**DOI:** 10.4103/0253-7613.45155

**Published:** 2008

**Authors:** Aylin Rezvani, Nihal Ozaras

**Affiliations:** Physical Medicine and Rehabilitation Department, Vakif Gureba Training Hospital, Istanbul, Turkey

**Keywords:** Ankylosing spondylitis, etanercept, infertility, TNF-α

## Abstract

The effect of TNF-α and TNF-α antagonists on semen quality in men is controversial. TNF-α levels are usually low in seminal plasma, but they tend to increase in inflammatory and infectious diseases. Etanercept is a highly-specific antagonist of TNF-α. In this report, we describe the development of pregnancy in a couple with a previously infertile husband, who received etanercept for ankylosing spondylitis.

## Introduction

TNF-α is a cytokine exerting a wide range of cellular effects. In testis, TNF-α is produced by germ cells and it has a role in the regulation of spermatogenesis.[1] TNF-α levels are usually low in seminal plasma, but they tend to increase in inflammatory and infectious diseases. Spermatozoa exposed to pathological concentrations of TNF-α can result in significant loss of their functional and genomic integrity.[2]

No studies measuring testicular TNF-α level in patients with ankylosing spondylitis (AS) have been found. We describe pregnancy development after etanercept administration to a husband with AS, in a couple with male factor infertility. It is postulated that increased levels of TNF-α in the testis might have decreased sperm production in this husband. TNF-α blockade by etanercept might have reversed this condition.

## Case Report

A 44-year-old man presented with a 20-year history of AS. The patient had morning stiffness of 45-minute duration and advanced deformities: kyphosis, decreased cervical and lumbar spinal mobility, 5 cm of chest-chin distance and 2 cm of chest expansion. He was prescribed a combination of sulphasalazine (2000 mg/day, p.o.) and indomethacin (100 mg/ day, p.o. as suppository), for the first 18-year of the illness. Two years ago, he discontinued this regimen due to active disease and side effects. He then received different analgesic drugs occasionally, during the last two years. On laboratory analysis, the erythrocyte sedimentation rate (ESR) and C-reactive protein (CRP) was 53 mm/hour and 2.78 mg/dL (normal: 0-0.8) respectively on February 1, 2005.

The patient was married for 20 years. The couple was evaluated for infertility and a diagnosis of “male factor infertility” was confirmed in 1996. They underwent *in vitro* fertilization in 1996 and 2000, but these attempts were unsuccessful. The husband's sperm concentrations were 2000 per milliliter in 1996 and 15000 per milliliter in 2000. He was receiving sulphasalazine during that time.

The patient was prescribed etanercept 25 mg twice weekly, s.c. On February 16, 2005, the first dose of the agent was injected. His wife, whose last menstruation date was March 5, 2005, had delayed menstruation. On gynecological examination on April 19, 2005 a 6-week gestation was detected. This spontaneous pregnancy developed after etanercept therapy, suggesting the role of etanercept in infertility. Semen analysis done on June 6, 2005 showed a sperm count of 140×10^6^ per milliliter concentration, 40% motility, 60% normal morphologic features. The measurements of ESR and CRP level were 35 mm/hour and 2.86 mg/dL respectively on March 16, 2005, and 38 mm/hour and < 0.86 mg/dL respectively on May 3, 2005.

## Discussion

The fertility status and sexual function of patients with AS were explored in several studies and controversial results were obtained.[3,4] Gordon *et al*. reported that there was no evidence of increased rate of infertility and no association between serum concentration of hormones and ESR.[3] On the other hand, Tapia-Serrano *et al*. evaluated testicular function with serum measurements of hormone levels in patients with AS and found abnormal results that correlated with disease activity.[4] On laboratory analysis, the erythrocyte sedimentation rate (ESR) and C-reactive protein (CRP) were measured as 53 mm/hour and 2.78 mg/dL (normal: 0.00-0.80) respectively on February 1, 2005. The measurements of ESR and CRP level were 35 mm/hour and 2.86 mg/dL respectively on March 16, 2005,

Semen analyses done in 1996 and 2000 revealed sperm concentrations that were in the subfertile ranges.[5] The patient was under sulphasalazine, a drug that was related to gross semen abnormalities and infertility in males, at that time.[6,7] The studies also reported that this negative effect of the drug on infertility was reversible and semen quality was dramatically improved after withdrawal of sulphasalazine for more than two months, resulting in successful pregnancies.[6–8] The patient discontinued the drug two years ago, but no pregnancy resulted even in the absence of protection. Hence sulphasalazine was probably not the cause of infertility in this case.

TNF-α is a potent cytokine produced by neutrophils, activated lymphocytes, natural killer cells, and activated macrophages.[9] In testis, TNF-α is secreted by germ cells and activated interstitial macrophages.[10,11] TNF-α levels are usually low in seminal plasma (< 10 pg/mL), but tend to increase in conditions such as inflammation.[12,13]

The effect of TNF-α and TNF-α antagonists, such as infliximab, on semen quality in men is controversial. A study revealed that TNF-α effectively inhibited apoptosis of human germ cells; but no significant effect of TNF-α on the activation of transcription factor, which is considered to be a mediator of TNF-α -induced survival signals, were observed.[1] Another study exploring the effects of the TNF-α in the rat seminiferous epithelium showed that TNF-α promotes cell survival and this prosurvival effect can be blocked by infliximab.[14] Mahadevan *et al.* reported that infliximab infusion did not affect the semen volume, sperm concentration, and forward progression, but it decreased sperm motility and percent of normal oval forms.[15]

On the other hand, Said *et al*. incubated sperm suspensions with different doses of TNF-α, TNF-α plus infliximab and only infliximab. Spermatozoa quality declined following incubation with TNF-α in a dose- and time-dependent manner. Sperm motility and membrane integrity were higher in the samples incubated with TNF-α plus infliximab than in the samples treated with TNF-α or infliximab alone. This study demonstrated that exposing spermatozoa to pathological concentrations of TNF-α can result in significant loss of their functional and genomic integrity and that infliximab is capable of reversing the toxic effects induced by TNF-α.[2]
